# Spatial Distribution and Factors Associated With Risky Health Behavior Among Adult Males Aged 15–59 Years in Ethiopia: Generalized Structural Equation Modeling

**DOI:** 10.3389/fpsyt.2022.688336

**Published:** 2022-03-15

**Authors:** Sewnet Adem Kebede, Adisu Birhanu Weldesenbet, Biruk Shalmeno Tusa

**Affiliations:** ^1^Department of Epidemiology and Biostatistics, Institute of Public Health, College of Medicine and Health Sciences, University of Gondar, Gondar, Ethiopia; ^2^Department of Epidemiology and Biostatistics, College of Health and Medical Sciences, Haramaya University, Haramaya, Ethiopia

**Keywords:** risky health behavior, spatial analysis, generalized structural equation modeling, Ethiopia, males

## Abstract

**Background:**

Alcohol drinking and tobacco smoking are the largest preventable causes of death and important risk factors for a number of non-communicable diseases and cause premature death and many socioeconomic consequences. Therefore, the present study is aimed to assess the spatial distribution of risky health behavior and its associated factors among adult males in Ethiopia.

**Methods:**

All men (12,688) within the age range of 15–59 years were included in the final analysis. The distribution of risky health behavior across the country was observed by using ArcGIS software. In SaTScan software, the Bernoulli model was fitted by Kulldorff methods to identify the purely spatial clusters of risky health behavior. Generalized Structural Equation Model (GSEM) was used to determine factors associated with risky health behavior (regular alcohol drinking and tobacco smoking).

**Results:**

Risky health behavior had spatial variation across the country. The primary clusters were located in Tigray, Amhara, and north-eastern Benishangul national regional states. Spatial scan statistics identified 118 primary clusters [Log-Likelihood ratio (LLR) = 524.8, *p* < 0.001]. Residence, frequency of listening to a radio, occupation, and frequency of watching television were significantly associated with drinking alcohol, whereas wealth index was associated with tobacco smoking. Age, region, educational status, marital status, and religion had association with both domains of risky health behavior.

**Conclusion:**

Risky health behavior had spatial variation across the country. Bans on advertising and promotion of alcohol and tobacco on national press media should be strengthened. Aggressive health education efforts should be directed toward this high-risk population (Tigray, Amhara, and north-eastern Benishangul regional states). Improving risky health behavior is an important approach to reducing health disparities and promoting a more cost-effective utilization of scarce resources in the public health sector.

## Background

Risky health behavior is a frequent or intense activity, which increases the risk of disease or injury now or in the future (1, 2). Risky health behaviors consist of inadequate physical activity, unhealthy dietary behavior, tobacco use, drug abuse, unprotected sexual practices, and harmful alcohol consumption (3).

On the ongoing strategies to prevent non-communicable diseases (NCD), both tobacco use and alcohol drinking are a serious challenge. Non-communicable diseases include cancer, diabetes, cardiovascular disease, and chronic obstructive pulmonary disease (COPD), which account for over 80% of all premature NCD deaths worldwide (4–6). All age groups (children, adults, and the elderly) are susceptible to the risk factors contributing to NCDs, whether from unhealthy dietary behavior, inadequate physical activity, exposure to tobacco smoke, or the harmful use of alcohol (7).

Sustainable development goals (SDG), which are related both to the health sector [SDGs 1 (No poverty), SDGs 2 (Zero hunger), SDGs 4 (Quality education), SDGs 5 (Gender education), and SDGs 6 (Clean water sanitation)] and outside the health sector [SDGs 8 (Decent work and economic growth), SDGs 10 (Reducing inequality), SDGs 11 (sustainable cities and communities), SDGs 16 (Peace, justice and strong institution), and SDGs 17 (Partnership for the goals)] are affected by risky health behavior, and more specifically it affects SDG3, which includes maternal mortality reduction, ability to fight communicable diseases, reduction of mortality from NCD, promote mental health, achieve universal health coverage, reduce injuries, and poisonings. Alcohol drinking and tobacco smoking are specifically stated under health Target 3.5: “strengthen the prevention and treatment of substance use, including narcotic drug abuse and harmful use of alcohol” (5, 8).

Globally, 5.3% of all deaths were attributed to harmful use of alcohol. Mortality due to a combination of disease (tuberculosis, HIV/AIDS, and diabetes) is lower than death due to alcohol consumption. Worldwide, alcohol was responsible for 7.2% (among persons 69 years of age and younger) of all premature mortality in 2016 (5, 7, 9). Recent World Health Organization (WHO) estimates indicate that about 1.1 billion people use tobacco worldwide. Seven million deaths with over 80% mortality taking place in low and mid-income countries is attributed to tobacco use (10).

The WHO global report showed that the prevalence of tobacco smoking is decreasing in almost all regions of the world, except African and Eastern Mediterranean regions, where the trends are flat (11). The highest number of deaths was recorded in Eastern Africa while the lowest was recorded in Central Africa (12). Although the highest levels of alcohol consumption are in Europe, Africa bears the heaviest burden of disease and injury attributed to alcohol (5).

Despite the fact that both men and women drink alcohol and tobacco, males consume more of both and are responsible for more alcohol and tobacco-related harm to themselves and others. The alcohol attributable burden of disease was higher among men than among women (13). Men smoke more tobacco than women in different societies and cultures (14–16).

Alcohol and tobacco use among adult men in Ethiopia was 46.6 and 7.3% in 2015, respectively (17). Even though fewer men die from tobacco in Ethiopia than the average in countries with low human development index, tobacco kills 258 men every week (18). Also, tobacco use directly fuels poverty. It drives resources from other basic needs such as nutrition, education, and health (8). The economic cost of smoking in Ethiopia amounts to 1,391 million Ethiopian birr (ETB), US$43.6 million. This include both direct and indirect costs due to early death and disease (18).

Different studies were conducted in different parts of Ethiopia using a univariate analysis to determine factors associated with each category of risky health behavior (19–22). In univariate analysis only one outcome variable is allowed. However, risky health behavior has two domains (alcohol drinking and tobacco smoking) which need to be considered as independent variables. Furthermore, the prevalence of risky health behavior is not geographically homogenous.

Thus, the present study applies spatial analysis to identify geographic distribution of risky health behavior and generalized structural equation model (GSEM) to determine factors associated with two domains of risky health behavior. Understanding the spatial distribution of alcohol drinking and tobacco smoking, along with the factors contributing to their use, is crucial to identify priority areas for developing targeted tobacco and alcohol control policies and reducing alcohol and tobacco related harm.

## Method

### Study Design, Period, and Setting

Population-based cross-sectional study was conducted in Ethiopia. The data was collected from January 18, 2016, to June 27, 2016. Administratively Ethiopia is divided into nine regional states and two city administrations. The regions are subdivided into zones which are further subdivided into woredas. Woredas are subdivided into the lowest administrative units called kebeles, and kebeles form Enumeration Areas (EAs) which represent primary sampling unit for EDHS data.

### Data Source and Extraction

The 2016 Ethiopian demographic and health survey men's data set was used to extract the dependent variable and important independent factors. The 2016 EDHS is the fourth survey implemented by the Central Statistical Agency (CSA) in collaboration with Federal Ministry of Health (FMoH) and the Ethiopian Public Health Institute (EPHI). It is nationally representative household surveys that provide data for a wide range of monitoring and impact evaluation indicators in the areas of population, health, and nutrition. The data sets were downloaded in Stata format with permission from the DHS program official database (http://www.dhsprogram.com) after an online request was made explaining the aim of the study.

### Study Population and Sampling Procedure

The study population was all men within age range of 15–59 years, and those men who are within the randomly selected EAs were study subjects. The 2016 EDHS sample was stratified and selected in two stages. In the first stage, a total of 645 Enumeration Areas (EAs) were selected with probability proportional to EA size and with independent selection in each sampling stratum. In the second stage of selection, a fixed number of households per cluster were selected with an equal probability systematic selection from the newly created household listing. The detailed sampling procedure was presented in the full 2016 EDHS report (23).

### Study Variables

The outcome variables with important predictors were extracted from Ethiopia Demographic and Health Surveys men's data set. Risky health behavior among men aged 15–59 was used as a dependent variable. Risky health behavior involves 2 items: regular alcohol use (one or more drinks per week in the last 12 months) and/or regular tobacco use (used chewing tobacco every day or smoked cigarettes every day in the last 30 days) (24). Risky health behavior is defined as those that potentially expose people to harm, or significant risk of harm, which prevent them from reaching their potential in life and which can cause significant morbidity or mortality (25). Depending on different literature review, independent variables included in the analysis are described in Table 1.

**Table 1:** Description of explanatory variables used in the analysis.

**Variables**	**Description**	**Category**
Age (years)	Age of household member	15–19, 20–24, 25–29, 30–34, 40–44, 45–49, 50–54, and 55–59
Educational level	Highest educational level attained	No education, primary, secondary and higher
Sex	Sex of the household head (the person considered responsible for the household)	Male, female
Marital status	Never married, currently married and formerly married (It includes divorced, separated and widowed)	Never married, married, and divorced/separated/widowed
Residence	Type of place of residence	Urban and rural
Wealth status	It is a composite measure of a household's cumulative living standard	Poor, middle and rich
Region	Region	Tigray, afar, amhara, oromia, somalia, benishangul, SNNPR, gambella, harari, addis ababa, and dire dawa
Occupation	Occupation	Not working, professional, clerical, sales, agricultural, services, skilled manual, unskilled manual, and others
Religion	Religion	Orthodox, protestant, muslim, and other
Frequency of watching television	Frequency of television exposure	Not at all, less than once a week, and at least once a week
Frequency of listening to radio	Frequency of radio exposure	Not at all, less than once a week, and at least once a week

*SNNPR, Southern Nations, Nationalities, and Peoples' Region*.

### Data Processing and Analysis

Data were processed and analyzed using Stata 14, ArcGIS 10.3, and SaTScan 9.6 softwares. The data were weighted because the overall probability of selection of each household is not a constant. The representativeness of the survey, correction of the difference of the sample vs. the target population, and probabilities of selection and reliable statistical estimates was maintained by applying weighting. Generalized structural equation model (GSEM) was used to determine factors associated with each item of risky health behavior (regular alcohol drinking and tobacco smoking). Each domain of risky health behavior was a binary variable that was analyzed with Bernoulli family and a logit link function.

**Figure 1:**
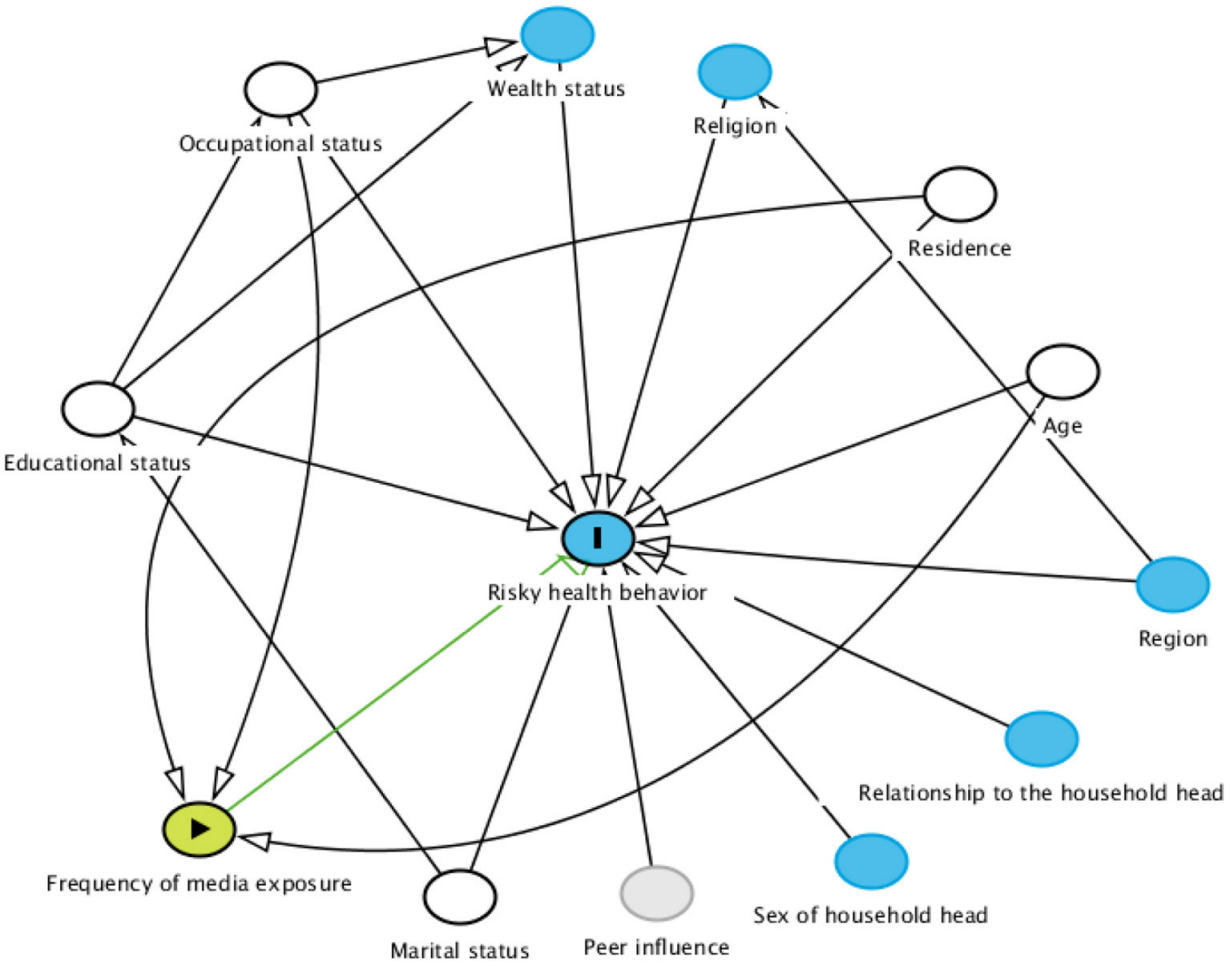
Hypothesized model for factors associated with risky health behavior among males in Ethiopia.

The postulated model in Figure 1 was used to start the analysis. By adding a path link, adjustments were taken interactively. At the end, an over identified model with minimum information criteria was taken.

A final model was retained based on statistical significance of path coefficient, the theoretical meaningfulness of the relationship, and minimum information criteria. Adjusted odds ratio (AOR) with a 95% confidence interval (CI) and *p*-value < 0.05 were declared as factors associated with risky health behavior.

#### Spatial Autocorrelation Analysis

To detect whether there is clustering of risky health behavior, spatial autocorrelation was conducted. Spatial autocorrelation based on feature locations and attribute values using the Global Moran's I statistic was calculated and Z-scores with corresponding *P*-values were presented. Statistically significant Moran's I index revealed the presence of overall nationwide clustering. Local cluster analysis was used for the confirmation of specific cluster areas.

#### Spatial Scan Statistical Analysis

Men aged 15–59 years without risky health behavior were taken as controls, and those with risky health behavior were taken as cases and represented by a 0/1 variable to fit the Bernoulli model and identify the geographical locations of statistically significant spatial clusters of risky health behavior among men using Kuldorff's SaTScan version 9.6 software. The number of cases in each location had Bernoulli distribution and the model required data with or without risky health behavior. A likelihood ratio test statistic was used to determine whether the number of observed cases within the potential cluster was significantly higher than the expected or not. Based on the 999 Monte Carlo replications primary and secondary clusters were identified using *p*-values and likelihood ratio tests (26).

## Results

### Characteristics of Study Population

A total of 12,688 men aged 15–59 were included in the analysis. About 10,187 (80.29%) of them were rural dwellers and 5,901 (46.51%) had primary education. The mean age of the participants was 30.83 years (SD ± 11.55 years). The household wealth index of 46.64% of the study subjects were in the two upper wealth quintiles (Table 2).

**Table 2:** Socio-demographic characteristics of respondents in Ethiopia, 2016 (*N* = 12,688).

**Variables**	**Weighted frequency**	**Percent**
**Age**		
15–19	2,572	20.27
20–24	1,883	14.84
25–29	1,977	15.58
30–34	1,635	12.88
35–39	1,385	10.92
40–44	1,206	9.51
45–49	947	7.47
50–54	582	4.59
55–59	501	3.95
**Residence**		
Urban	2,501	19.71
Rural	10,187	80.29
**Region**		
Tigray	795	6.27
Afar	90	0.71
Amhara	3,230	25.46
Oromia	4,758	37.50
Somali	329	2.59
Benishangul	128	1.00
SNNPR	2,596	20.46
Gambela	37	0.29
Harari	31	0.25
Addis ababa	621	4.90
Dire dawa	73	0.57
**Level of education**		
No education	3,840	30.26
Primary	5,901	46.51
Secondary	1,846	14.55
Higher	1,101	8.67
**Religion**		
Orthodox	5,690	44.84
Protestant	2,748	21.66
Muslim	3,985	31.41
Other^*^	265	2.09
**Sex of household head**		
Male	11,122	87.66
Female	1,566	12.34
**Wealth index**		
Poorest	2,009	15.83
Poorer	2,318	18.27
Middle	2,443	19.25
Richer	2,727	21.49
Richest	3,191	25.15
**Marital status**		
Never married	4,895	38.58
Married/living together	7,471	58.88
Divorced/separated/widowed	322	2.54
**Currently working**		
No	1,430	11.27
Yes	11,258	88.73

Others^*^= Catholic, cultural belief; SNNPR, Southern Nation Nationalities and Peoples Region.

### Spatial Distribution of Risky Health Behavior

**Figure 2:**
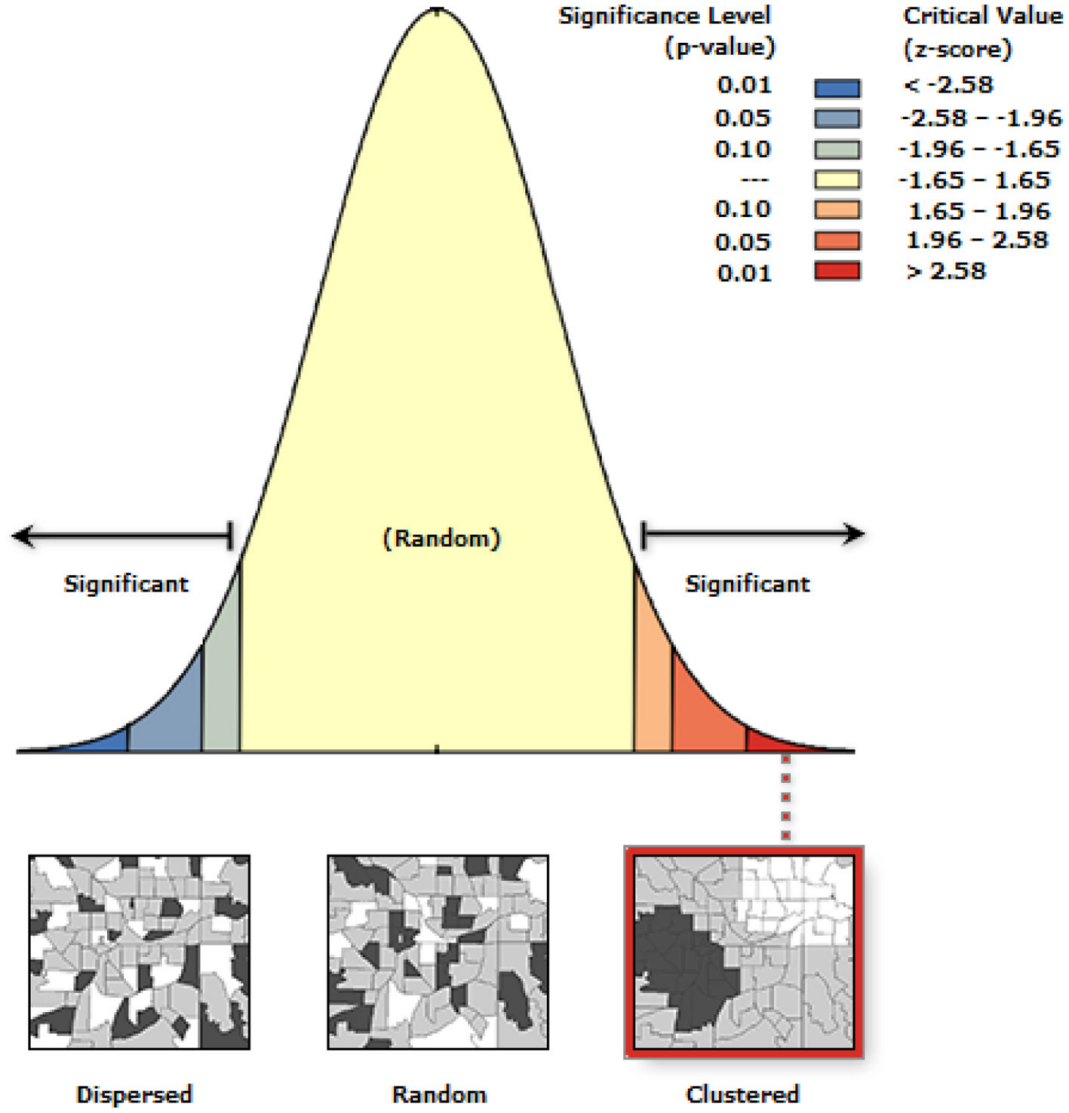
Spatial autocorrelation analysis of risky health behavior among males in Ethiopia, 2016.

The spatial distribution of risky health behavior was found to be spatially clustered in Ethiopia with Global Moran's I 0.12 (*p* < 0.0001). Cluster of high rates in risky health behavior was observed in the study area. The outputs were automatically generated keys given the z-score of 4.02 indicated that there is <1% likelihood that this clustered pattern could be the result of a random chance. The bright red and blue colors to the end tails indicates an increased significance level (Figure 2).

**Figure 3:**
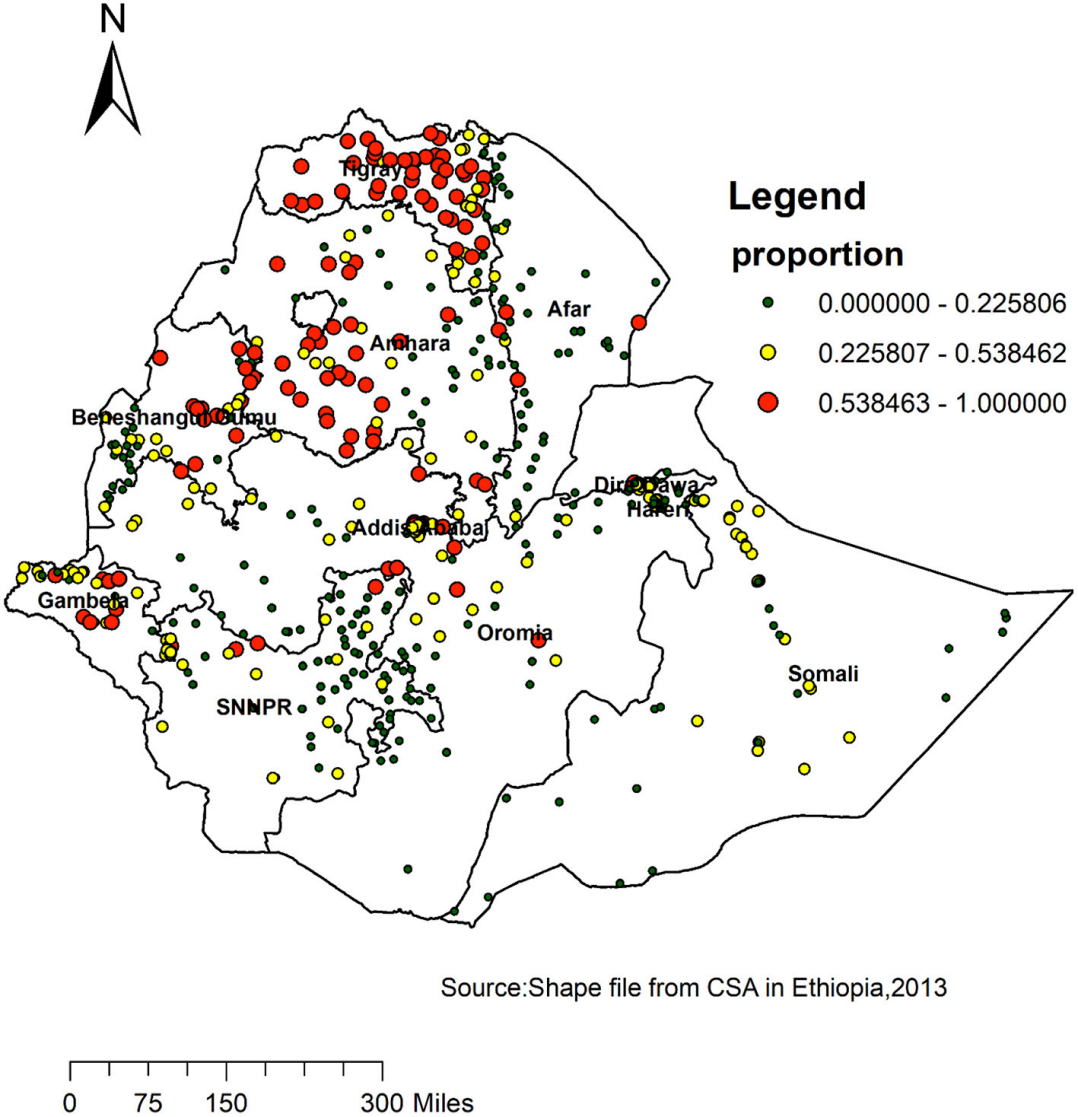
Spatial distribution of risky health behavior among adult males across regions in Ethiopia, 2016.

Spatial clustering of risky health behavior was found at regional levels. The overall prevalence of risky health behavior in men was 31% in one or both of the domains such as regular tobacco smoking and regular alcohol drinking. The highest risky health behavior was spatially clustered in Tigray, Amhara, eastern part of Benishangul and Gambella regions; Oromia and Somali regions had lowest risky health behavior (Figure 3).

### Spatial SaTScan Analysis of Risky Health Behavior (Bernoulli Based Model)

A total of 125 significant clusters were identified, of which 118 most primary clusters and 7 secondary clusters of risky health behavior were identified. The primary clusters were located in Tigray, Amhara, and north-eastern Benishangul regional states. The primary clusters were centered at 12.669915 N, 36.775082 E, with 313.13 km radius, a relative risk (RR) of 2.41, and Log-Likelihood ratio (LLR) of 524.8, at *p* < 0.001. It showed that men within this area are at 2.4 times higher risk for risky health behavior than men outside the area (Table 3).

**Table 3:** Significant spatial clusters with high-rate risky health behavior among males in Ethiopia, 2016.

**Cluster**	**Enumeration area (cluster) identified**	**Coordinate (radius)**	**Population**	**Case**	**RR**	**LLR**	***P*-value**
1	279,292,638,640,52,296,504,312,327,612,163,322,152, 169,259,158,431,73,602,415,516,512,80,258,361,253, 541,628,386,132,382,548,167,199,515,456,425,188,615, 498,109,429,403,583,340,24,3,533,551,268,246,98, 627, 559,181,255,256,120,38,528,36,584,542,78,66,156, 375, 579,150,636,137,183,575,545,597,474,35,364,400,590, 244,81,494,538,206,300,184,401,424,392,136,591,176, 355,84,482,143,531,481,478,430,160,45,604,229,449, 97,237,461,94,550,351,218,200,442,479,605,350	(12.669915N, 36.775082 E)/313.13 km	2,599	1,518	2.41	524.8	<0.001
2	106, 105, 221, 231, 549, 291, 469	(8.211902 N, 34.451017 E)/16.65 km	127	77	1.95	23.2	<0.001

*RR, Relative risk; LLR, Log Likelihood Ratio*.

**Figure 4:**
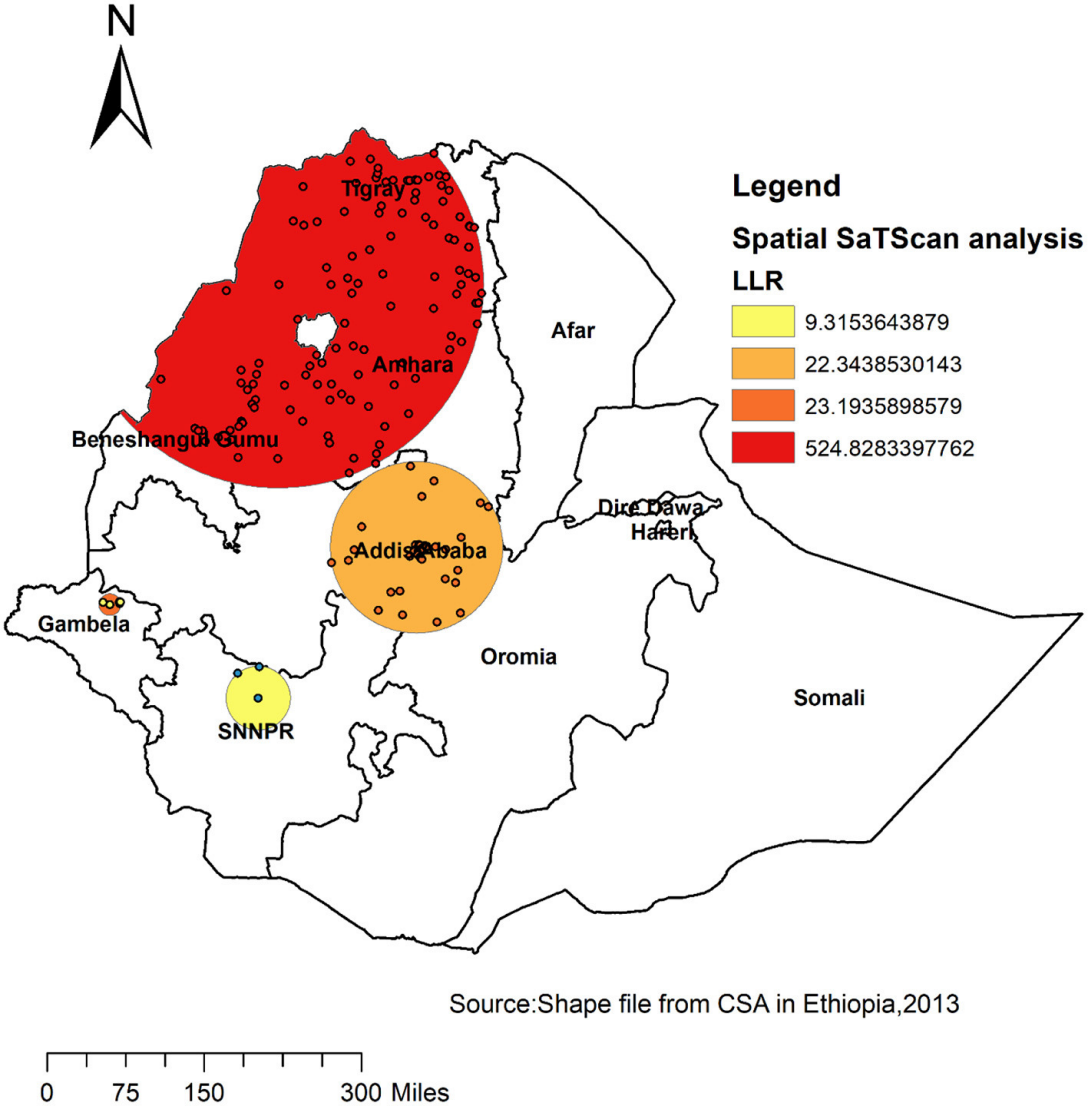
Primary and secondary clusters of risky health behavior among adult males across regions in Ethiopia, 2016.

The bright red color indicates that the most statistically significant spatial windows of risky health behavior. There was high risky health behavior within the cluster than outside the cluster (Figure 4).

### Factors Associated With Risky Health Behavior

**Figure 5:**
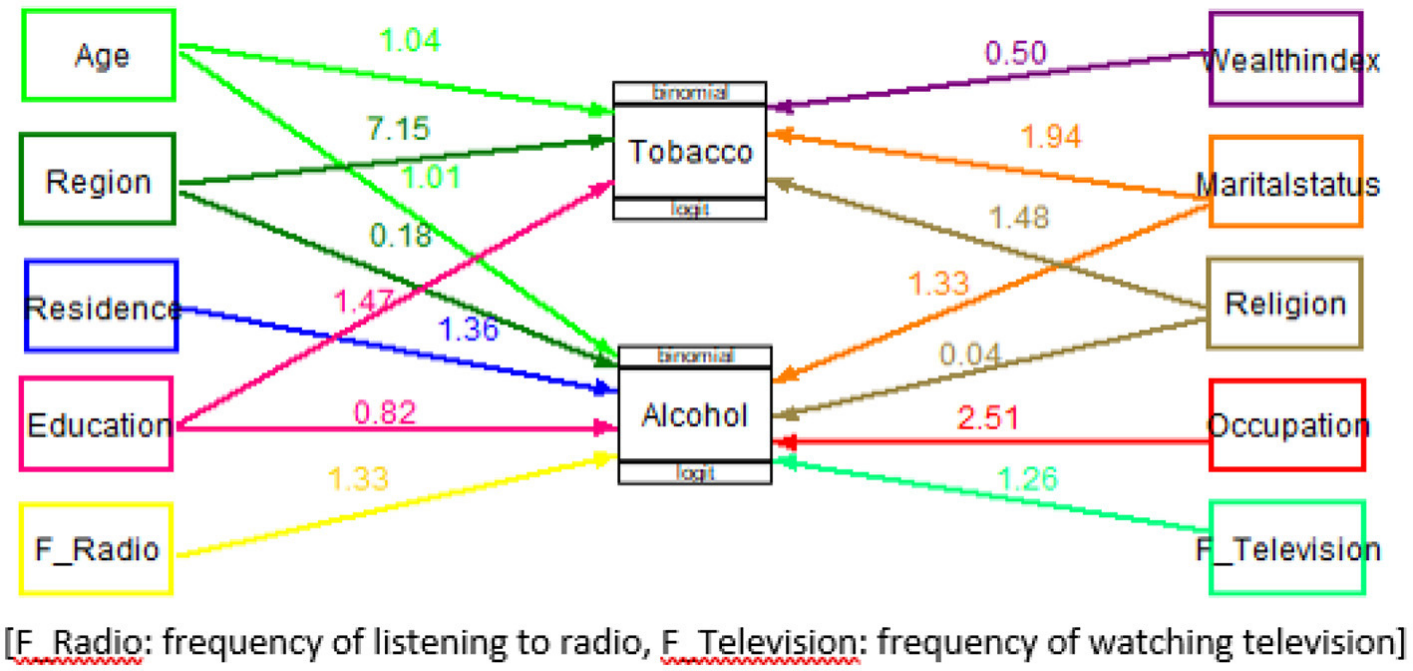
GSEM for associated factors of risky health behavior among adult males in Ethiopia, 2016.

The final model for associated factors of risky health behavior is shown in Figure 5 and Table 4. This model had included ten exogenous variables (age, region, residence, educational status, frequency of listening to radio, marital status, religion, occupation, wealth index, and frequency of watching television) and two endogenous variables (alcohol drinking and tobacco smoking). All path coefficients were statistically significant at an alpha level of 0.05. Variables, namely sex of the household head and relationship to the household head, were excluded from the final model as their effects were not statistically significant at an alpha level of 0.05.

**Table 4:** Associated factors of risky health behavior among adult males in Ethiopia, 2016.

**Variables**	**Alcohol drinking**		**Tobacco smoking**	
	**AOR**	**95% CI**	**AOR**	**95% CI**
**Age**				
15–29	Ref	Ref	Ref	Ref
30–44	1.42	(1.25, 1.62)	2.45	(2.02, 2.97)
45–59	1.34	(1.15, 1.56)	2.28	(1.81, 2.87)
**Region**				
Tigray	Ref	Ref	Ref	Ref
Afar	0.18	(0.11, 0.28)	7.15	(3.53, 14.46)
Amhara	0.51	(0.43, 0.61)	0.92	(0.40, 2.12)
Oromia	0.58	(0.48, 0.71)	4.99	(2.51, 9.89)
Somali	0.08	(0.04, 0.18)	17.44	(8.85, 34.32)
Benishangul	0.92	(0.74, 1.15)	13.02	(6.67, 25.43)
SNNPR	0.36	(0.29, 0.44)	2.81	(1.35, 5.87)
Gambela	0.87	(0.69, 1.09)	19.23	(9.71, 38.12)
Harari	0.18	(0.12, 0.25)	19.72	(9.91, 39.26)
Addis ababa	0.37	(0.30, 0.47)	10.13	(5.05, 20.28)
Dire dawa	0.28	(0.21, 0.38)	17.49	(8.85, 34.54)
**Level of education**				
No education	Ref	Ref	Ref	Ref
Primary	0.95	(0.83, 1.09)	1.35	(1.12, 1.64)
Secondary	0.82	(0.68, 0.98)	1.47	(1.13, 1.91)
Higher	0.83	(0.66, 1.05)	1.28	(0.96, 1.71)
**Watching television**				
Not at all	Ref	Ref	Ref	Ref
Less than once a week	1.26	(1.10, 1.45)	–	–
At least once a week	1.35	(1.15, 1.59)	–	–
**Listening to radio**				
Not at all	Ref	Ref	Ref	Ref
Less than once a week	1.33	(1.15, 1.53)	–	–
At least once a week	1.42	(1.25, 1.62)	–	–
**Marital status**				
Never married	Ref	Ref	Ref	Ref
Married/living together	1.33	(1.15, 1.54)	1.94	(1.54, 2.44)
Divorced/separated/widowed	1.79	(1.34, 2.39)	3.63	(2.49, 5.28)
**Religion**				
Orthodox	Ref	Ref	Ref	Ref
Protestant	0.12	(0.09, 0.14)	0.82	(0.60, 1.11)
Muslim	0.04	(0.03, 0.05)	1.84	(1.48, 2.29)
Other	0.40	(0.29, 0.55)	1.96	(1.23, 3.16)
**Occupation**				
Not working	Ref	Ref	Ref	Ref
Professional	2.51	(1.87,3.36)	–	–
Clerical	3.03	(1.91,4.82)	–	–
Sales	3.12	(2.36, 4.12)	–	–
Agricultural	2.68	(2.14,3.36)	–	–
Services	2.32	(1.63, 3.29)	–	–
Skilled manual	3.47	(2.70, 4.46)	–	–
Unskilled manual	3.05	(2.15,4.32)	–	–
Others	2.69	(2.04, 3.56)	–	–
**Wealth status**				
Poor	Ref	Ref	Ref	Ref
Middle	–	–	0.85	(0.67, 1.09)
Rich	–	–	0.50	(0.41, 0.61)
**Residence**				
Urban	Ref	Ref	Ref	Ref
Rural	1.36	(1.13, 1.64)	–	–

Others^*^= Catholic, cultural belief.

Men aged 30–44 and 45–59 years had the highest probability of smoking tobacco compared to those aged 15–29 [(AOR = 2.45, 95% CI = 2.02–2.97) (AOR = 2.28, 95% CI = 1.81–2.87)], respectively. The odds of drinking alcohol among men aged 30–44 and 45–59 years is 58 and 66% higher as compared with those aged 15–29.

The odds of drinking alcohol lower in Afar, Amhara, Oromia, Somali, SNNPR, Harari, Addis Ababa, and Dire Dawa as compared with Tigray. However, the probability of smoking tobacco higher in Afar, Oromia, Somali, Benishangul, SNNPR, Gambela, Harari, Addis Ababa, and Dire Dawa as compared with Tigray.

The odds of drinking alcohol are 18% lower among those men who completed secondary education as compared with those with no education. On the other hand, the likelihood of smoking tobacco is 35 and 47% higher among those who completed primary and secondary education, respectively, as compared with those with no education.

The odds of drinking alcohol are 26 and 35% higher among men who were watching television less than once a week and at least once a week, respectively, as compared with those who were not watching television at all.

The odds of drinking alcohol are 33 and 42% higher among men who were listening to radio less than once a week and at least once a week, respectively, as compared with those who were not listening to radio at all.

The likelihood of drinking alcohol is 33 and 79% higher among men who were married and separated as compared with those who were never married. Being married and separated were 1.94 times and 3.63 times more likely to smoke tobacco than those who were never married.

The odds of drinking alcohol are 88, 96, and 60% lower among Protestant, Muslim, and Catholic religion followers, respectively, as compared with Orthodox religion followers. The likelihood of smoking tobacco increased by 84% among men who were Muslim religion follower as compared with Orthodox religion followers.

Men with active employment were more likely to drink alcohol as compared with the unemployed. The probability of smoking tobacco decreased with wealth. The odds of smoking tobacco are 50% lower among those men with rich wealth index than those in poor wealth index. Men in rural areas were 1.36 times more likely to drink alcohol than those in urban areas.

## Discussion

Using the EDHS data and an appropriate modeling approach, this study further assessed spatial distribution and factors associated with risky health behavior (regular alcohol drinking and/or regular tobacco smoking) among men in Ethiopia. This study revealed that the spatial distribution of risky health behavior in Ethiopia was non-random.

The current study demonstrated a geographic difference of risky health behavior among 4 of the 11 Ethiopian regions. Risky health behavior was highly clustered in Tigray, Amhara, eastern part of Benishangul, and Gambella regional states of Ethiopia. In line with this, high proportion clustering, spatial scan statistics analysis revealed that 125 significant clusters were found. The geographical difference of risky health behavior across the regional states might be attributable to the regional variation of culture and religion, difference in climate, and number of people who were unemployed in the specified regions.

According to the final model age, region, residence, educational status, frequency of listening to radio, marital status, religion, occupation, wealth index, and frequency of watching television were identified as associated factors of risky health behavior in Ethiopia.

Men aged 30–44 and 45–59 years had a higher likelihood of risky health behavior compared with men aged 15–29 years. This finding is in line with other study finding from Zambia (27), Namibia (28), and India (29). This could be due to the fact that as age increases, they can afford the cost, and their job and family tension cause the individual to take smoking and drinking as coping mechanism. This finding is in contrast with the study done in Burkina Faso (30).

Like previous studies (31–33), in the current analysis, educated men were less likely to drink alcohol as compared to those with no education. It is true that education enhances awareness about the health effects of alcohol products and affects a person's cognitive functioning, having health oriented allocation of resource, make them more receptive to health education messages, or more able to communicate and access appropriate health services (34–36). Educated men were more likely to smoke tobacco as compared to those with no education. This findings are in contrast with the finding of the study done in Zambia (27). This could be due to a significant number of people who believe that smoking is stylish and more modern than drinking alcohol. This could be the reason for the discrepancy in drinking alcohol and smoking tobacco among educated men.

Those men who have occupation were more likely to drink alcohol as compared with those who have no occupation. This finding is in line with other studies (37, 38). This could be due to the fact that men with active employment had a higher level of socioeconomic status and low financial pressure. This finding is in contrast with study findings from France (31).

According to place of residence, there is a significant difference in the proportion of men who drink alcohol. The probability of drinking alcohol was higher for rural residents compared with urban residents. Level of exposure to alcohol and cultural difference could be the reason for this discrepancy. Furthermore, there may be social pressure to drink alcohol in rural residences.

The rich wealth index compared with poor wealth index was associated with a lower risk of smoking tobacco. Results of this study show that the odds of smoking tobacco are 50% lower among those men with rich wealth index than those in poor wealth index; this is in line with the results of other studies conducted in other countries such as Zambia (27) and India (39). Being responsiveness to health promotion and health information about risky health behavior might be high among high income groups.

In our study, religion was significantly associated with risky health behavior. Non-orthodox participants were at low risk of drinking alcohol compared with orthodox participants. On the contrary, non-orthodox men were at high risk of smoking tobacco compared with orthodox participants. This could be due to the fact that non-orthodox participants located in these areas cultivate tobacco plants and prepare for local markets. The married and divorced men had a higher likelihood of risky health behavior compared with those who have never been married. This could be due to the fact that married and divorced men had emotional distress that may lead them to become smoker or drinker for comfort.

Exposure to mass media at least once a week increased the risk of alcohol drinking compared to being not exposed to mass media at all. This findings are in contrast with the finding of the study done in Zambia (27). This could be due to the fact that those who watched television or listened to the radio were exposed to alcohol industry advertisement as possible causal agents in the stimulation of demand for alcohol.

One of the strengths of this study was using a large sample size, which is representative at national and regional levels, providing depths for generalization the target population of Ethiopia. Furthermore, this paper builds a body of knowledge on the spatial disparity of risky health behavior and identifies hotspot areas by simultaneous use of both ArcGIS and Sat Scan statistical tests to enhance decision making on public health problem of risky health behavior through cluster analysis.

This study is not free from limitation. It is challenging to know the exact cases' location since the location of data values was shifted 1–2 km for urban and 5 km for rural areas for data confidentiality issues. Similarly, essential factors such as peer pressure to smoke or drink, factors to assess other domains of risky health behavior like physical inactivity and unhealthy diet were not available in the EDHS so that it was not possible to incorporate these variables in the analysis. Furthermore, alcohol and tobacco use were self-reported by the survey participants, hence there is a chance of underreporting.

## Conclusion

This study indicated that considerable geographic disparities in risky health behavior occur within Ethiopia. The results of this study revealed that risky health behavior among men varied across the country; significant risky health behavior hotspots were observed in Tigray, Amhara, and north-eastern Benishangul national regional states. Residence, frequency of listening to a radio, occupation, and frequency of watching television were significantly associated with drinking alcohol, while wealth index was associated with tobacco smoking. Age, region, educational status, marital status, and religion had a significant association with both domains of risky health behavior. The national press media should involve in ban of advertising and promotion of alcohol and tobacco. Populations living in recognized hotspot areas should be targeted for aggressive health education. Improving risky health behaviors is an important approach to reducing health disparities and for more cost-effective utilization of scares resources in the public health sector.

## Data Availability Statement

The original contributions presented in the study are included in the article/supplementary material, further inquiries can be directed to the corresponding author/s.

## Ethics Statement

The study doesn't involve the collection of information from subjects. Consent to participate is not applicable. We sent a one-page proposal abstract of the study to the DHS program office. They gave permission to access the data with reference number of 144751.

## Author Contributions

Conception and design of the work, acquisition of data, analysis, and interpretation of data was done by SK. Data curation, drafting the article, revising it critically for intellectual content, validation, and final approval of the version to be published was done by SK, AW, and BT. All authors have read and approved the final manuscript.

## Conflict of Interest

The authors declare that the research was conducted in the absence of any commercial or financial relationships that could be construed as a potential conflict of interest.

## Publisher's Note

All claims expressed in this article are solely those of the authors and do not necessarily represent those of their affiliated organizations, or those of the publisher, the editors and the reviewers. Any product that may be evaluated in this article, or claim that may be made by its manufacturer, is not guaranteed or endorsed by the publisher.

## Abbreviations

AOR: adjusted odds ratio
CI: confidence interval
COPD: chronic obstructive pulmonary disease
CSA: central statistics agency
EA: enumeration area
EDHS: Ethiopian demographic and health survey
GSEM: generalized structural equation model
SD: standard deviation
SDG: sustainable development goal
SNNPR: southern nation and nationality and peoples region
WHO: world health organization.
